# S08-1: How can we scale up local physical activity policy monitoring initiatives?

**DOI:** 10.1093/eurpub/ckae114.232

**Published:** 2024-09-26

**Authors:** Sven Messing, Aurélie Van Hoye, Karim Abu-Omar, Peter Gelius, Olivier Riquier, Antonina Tcymbal, Benjamin Tezier

**Affiliations:** Friedrich-Alexander-Universität Erlangen-Nürnberg, Department of Sport Science and Sport, Erlange, Germany; Physical Activity for Health research group, Health Research Institute, Physical Education and Sport Sciences department, Ireland; Physical Activity for Health research group, Health Research Institute, Physical Education and Sport Sciences department, Ireland; UMR1319 INSPIRE, Université de Lorraine; Friedrich-Alexander-Universität Erlangen-Nürnberg, Department of Sport Science and Sport, Erlange, Germany; Institut des Sciences du Sport, Université de Lausanne; UMR1319 INSPIRE, Université de Lorraine; LAHMESS, Université Côte d‘Azur; Friedrich-Alexander-Universität Erlangen-Nürnberg, Department of Sport Science and Sport, Erlange, Germany; UMR1319 INSPIRE, Université de Lorraine

## Abstract

**Purpose:**

There is a growing body of evidence on the effectiveness of physical activity (PA) policies, and numerous tools have been developed to monitor such policies. However, a recent literature review revealed that only a limited number of tools focus on PA policy monitoring at the local level. While some of these tools focus on an in-depth data collection in single municipalities, there are only selected examples of large-scale policy monitoring initiatives. This raises the question how to scale up local physical activity policy monitoring initiatives in different countries and considering local contexts.

**Methods:**

To generate knowledge on local PA policy monitoring, a qualitative iterative research design was adopted, including (a) the mapping of indicators of established local PA policy monitoring tools and (b) a co-construction process with policy-makers in France and Germany. The co-construction process with local level policy-makers from different sectors generated data on facilitators and barriers for large-scale policy monitoring, and helped to design a preliminary strategy for scaling up PA policy monitoring at local level.

**Results:**

The comparison of established tools provided a systematic overview of indicators and processes used to monitor local PA policy. Based on the co-construction process, a shortlist of indicators with a high relevance for the involved municipalities was created, considering the perspective of policy-makers from different sectors and countries. The preliminary strategy for scaling up local PA policy monitoring initiatives addresses the two aspects of increasing the reach (number of municipalities involved) and frequency of respective initiatives.

**Conclusions:**

The results show that a strategy for scaling up local PA policy monitoring can be developed based on a co-construction process with policymakers. However, only a limited number of municipalities was involved in this explorative study. Further research is needed to validate the findings for France and Germany, and to test the transferability of this strategy to other countries.

**Funding Source:**

This project has received funding by BayFrance under the grant agreement number FK-06-23.

